# In Vitro Maturation, In Vitro Oogenesis, and Ovarian Longevity

**DOI:** 10.1007/s43032-023-01427-1

**Published:** 2023-12-30

**Authors:** Sherman J. Silber, Sierra Goldsmith, Leilani Castleman, Katsuhiko Hayashi

**Affiliations:** 1 Infertility Center of St. Louis at St. Luke’s Hospital, St. Louis, MO 63017 USA; 2 Department of Genome Biology, Graduate School of Medicine, Osaka University, Osaka, 565-0871 Japan

**Keywords:** Cancer and Fertility, In-Vitro Oocyte Maturation, Ovary Tissue Cryopreservation, Ovary Transplantation, Primordial Follicle Recruitment

## Abstract

This paper will review a remarkable new approach to in vitro maturation “IVM” of oocytes from ovarian tissue, based on our results with in vitro oogenesis from somatic cells. As an aside benefit we also have derived a better understanding of ovarian longevity from ovary transplant. We have found that primordial follicle recruitment is triggered by tissue pressure gradients. Increased pressure holds the follicle in meiotic arrest and prevents recruitment. Therefore recruitment occurs first in the least dense inner tissue of the cortico-medullary junction. Many oocytes can be obtained from human ovarian tissue and mature to metaphase 2 in vitro with no need for ovarian stimulation. Ovarian stimulation may only be necessary for removing the oocyte from the ovary, but this can also be accomplished by simple dissection at the time of ovary tissue cryopreservation. By using surgical dissection of the removed ovary, rather than a needle stick, we can obtain many oocytes from very small follicles not visible with ultrasound. A clearer understanding of ovarian function has come from in vitro oogenesis experiments, and that explains why IVM has now become so simple and robust. Tissue pressure (and just a few “core genes” in the mouse) direct primordial follicle recruitment and development to mature oocyte, and therefore also control ovarian longevity. There are three distinct phases to oocyte development both in vitro and in vivo: in vitro differentiation “IVD” which is not gonadotropin sensitive (the longest phase), in vitro gonadotropin sensitivity “IVG” which is the phase of gonadotropin stimulation to prepare for meiotic competence, and IVM to metaphase II. On any given day 35% of GVs in ovarian tissue have already undergone “IVD” and “IVG” in vivo, and therefore are ready for IVM.

## Introduction

Two objectives for improving preservation of fertility in cancer patients are IVM (in vitro maturation) of oocytes from ovarian tissue, and IVG (in vitro gametogenesis) of oocytes from somatic cells transformed into stem cells. Those two objectives are now clinically within reach, and both reinforce each other.

For IVM, studies in the last decade from Belgium and Denmark, more recently the USA, have shown that GV (germinal vesicle) oocytes retrieved from ovarian tissue dissection (not via ultrasound guided needle aspiration) can be readily cultured to M2 (metaphase 2) mature, functional oocytes [1–4]. Furthermore, these in vitro cultured M 2 oocytes have resulted in live births [3, 5–7].

For IVG, in mice, somatic cells have been transformed into functional M 2 oocytes leading to live births [8–16]. In humans normal PGCs (primordial germ cells) have been generated from skin biopsy in azoospermic men and in post menopausal women over 50 years of age [8–20]. It may be only a matter of time before these human PGCs can be fully transformed into functional oocytes. Skin cells have also been transformed into PGCs in endangered species in order to eventually resurrect them. The Northern white rhino is now extinct, but their skin cells have been transformed into beautiful IPS cells and PGCs [21].

**Fig. 1:**
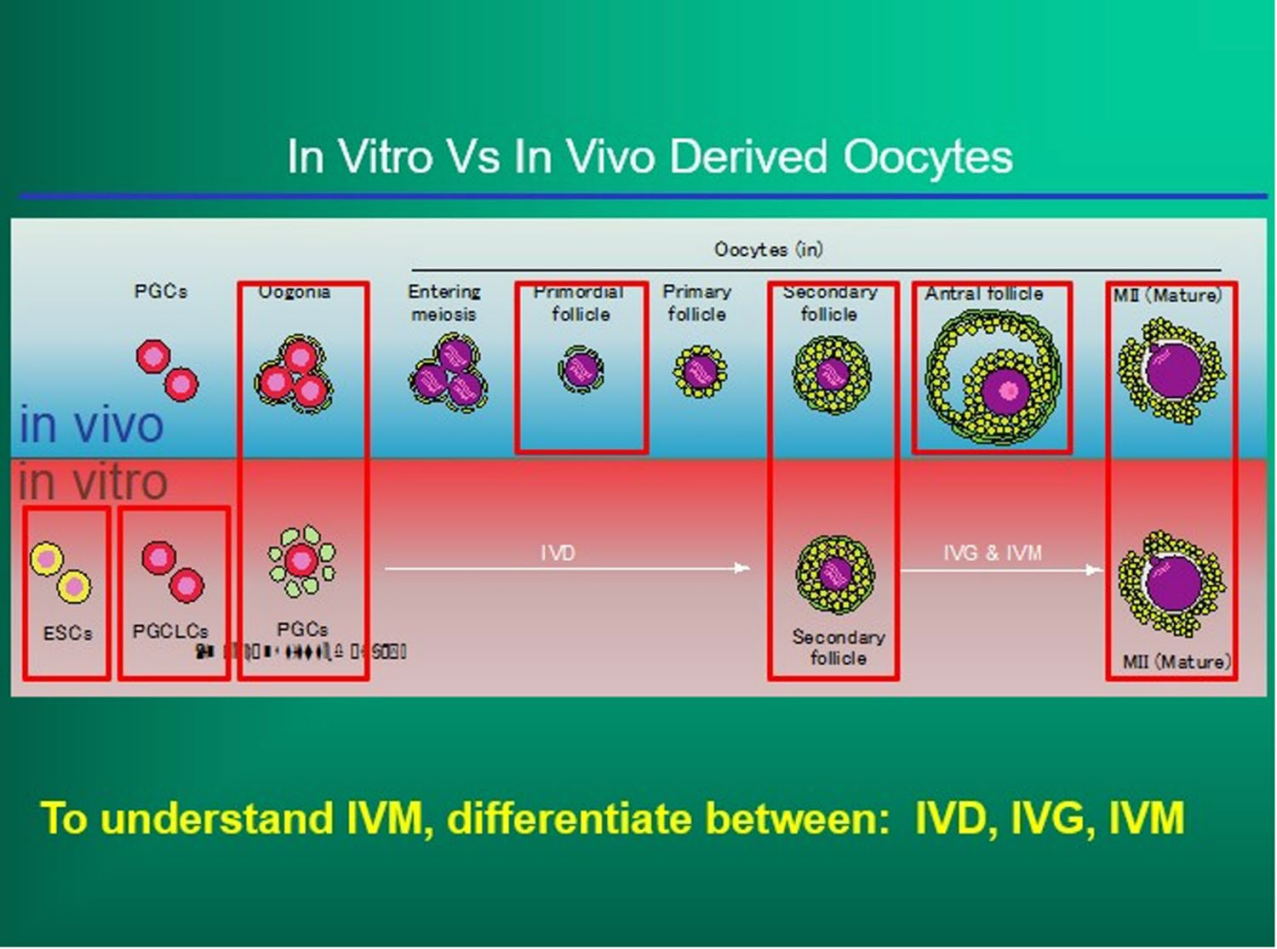
Comparison of in vivo oogenesis to in vitro oogenesis from stem cells [11]

The success of in vitro gametogenesis in generating live offspring from somatic cell transformation has helped to elucidate the mechanism controlling primordial follicle arrest, recruitment, and ovarian longevity [12, 14, 22, 23]. In mice, using eight “core genes” and low pressure, primordial follicles can be unlocked and cultured to M 2 oocytes. Furthermore, in mice, fetal granulosa cells can also be produced from adult skin cells transformed into IPS cells (stem cells). Fetal, not adult, granulosa cells are necessary to convert PGCs into complete oocytes. These “manufactured” fetal granulosa cells can then be used to produce competent M 2 oocytes from already produced PGCL cells (PGCs) in mice [13]. Since PGCs have been robustly produced now in humans from skin biopsy cells, all that will be needed to produce competent M 2 oocytes in adult humans from skin biopsy is to find the species specific genes in the human (using Yoshino et al methods) that convert skin cells to fetal granulosa cells [13]. Even now, we can use this clearer knowledge of ovarian-oocyte physiology to simplify greatly the use of IVM for fertility preservation in humans from ovarian tissue (Fig. 1).

## In vitro Maturation Of Oocytes from Ovarian Tissue

It is clinically not yet possible to attempt the task of maturing human primordial follicles in vitro. However, it will be possible in the future utilizing the “eight core genes” approach [14]. But at present, it is simpler and indeed quite robust just to perform IVM on GV oocytes that have already been prepared “naturally” for maturation in vivo (by “IVD” and “IVG” phases). This simple approach arose out of our efforts in the mouse to transform skin cells to functional oocytes (in vitro gametogenesis), which has solved with clarity some of the mysteries of ovarian function. The problem in the past was doing too many steps that hindered rather than helped results. A clearer understanding of ovarian function and of in vitro gametogenesis explains the great simplicity of IVM.

**Fig. 2:**
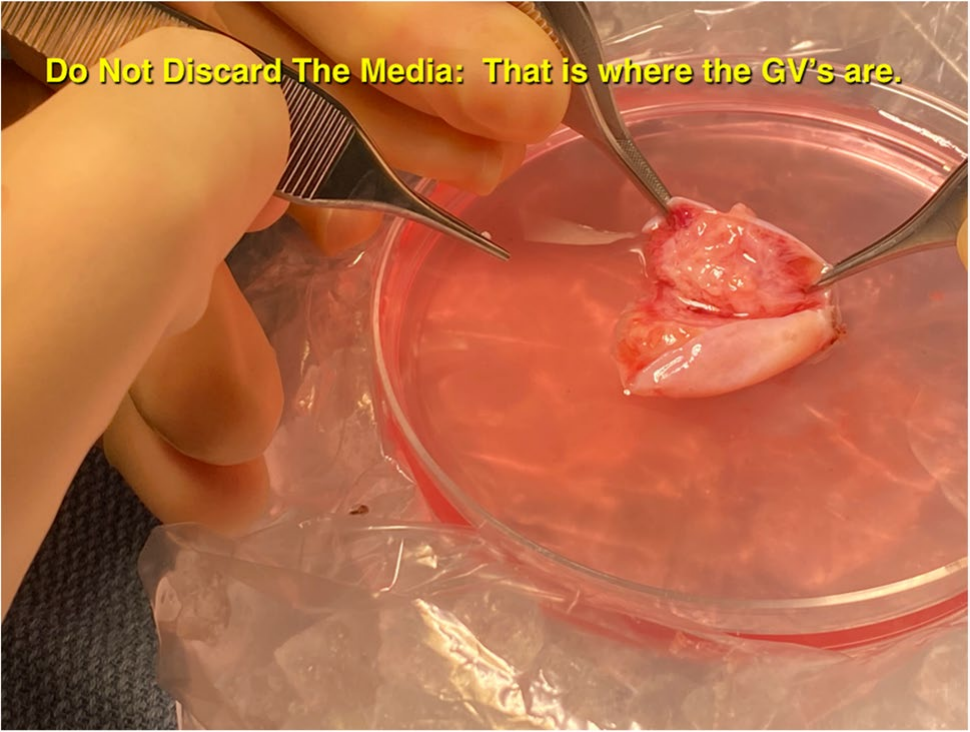
Dissection of ovarian cortex from the medulla

**Fig 3:**
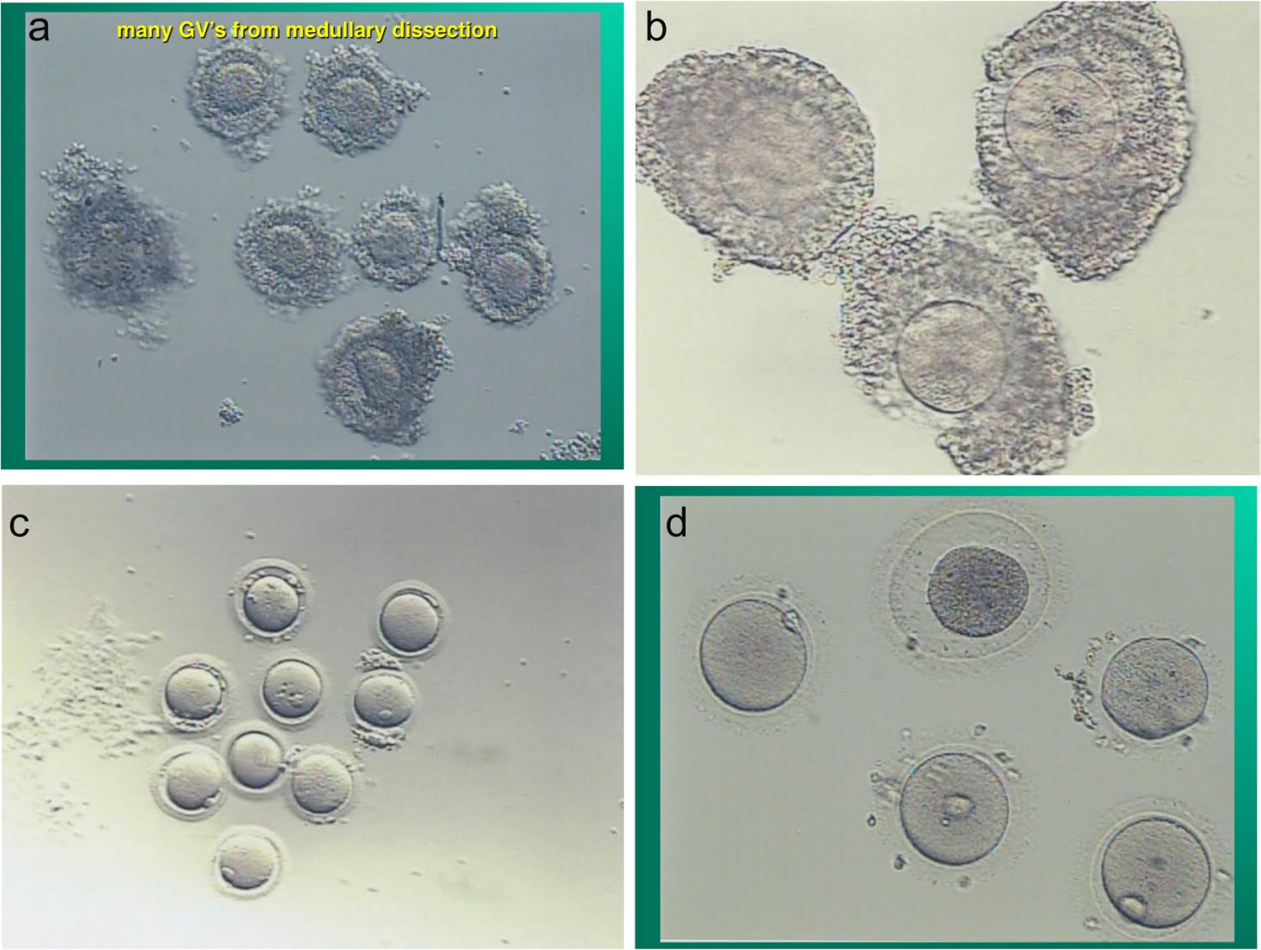
**a** Day 0 Compact appearance of cumulus complexes (CCs) at time of dissection [4]. **b** Day 1 Spreading of cumulus complexes after 24 h in culture with HCG or LH [4]. **c** Day 1 Metaphase II oocytes after 24 h in culture with HCG or LH [4]. **d** Three normal M II oocytes, one M I oocyte, and one degenerated oocyte, resulting from cultured ovarian tissue GVs, on day 2 [4]

It is much easier to obtain many GVs from dissection then needle aspiration. That is one key to the robustness of IVM. Do not interfere with natural meiotic competence by ovarian stimulation which only is useful for follicle enlargement. At the time of oophorectomy for ovarian tissue cryopreservation, when the ovarian cortex has been dissected from the medulla (Fig. 2) and divided into slices for cryopreservation, the “spent” medium in which the dissection took place should be examined for many free, loose cumulus complexes, which usually contain immature germinal vesicle oocytes [4]. These cumulus complexes are then placed in culture with widely varying concentrations of FSH and human chorionic gonadotrophin (HCG) or LH. The cumulus is stripped between 24 and 44 h later (Fig. 3a–d). The reason that a variety of media and gonadotrophin concentrations were employed in our center is based on our previously published data from in-vitro gametogenesis in mice [8–12, 14]. We wished to see whether IVM could proceed in a variety of various media because the oocytes have already been able to develop meiotic competence from in vivo “IVD” and IVG” (Fig. 1). IVD is the phase of primordial follicle development (3 weeks in mouse; 4.5 months in human) that is inert to gonadotropin. IVG in this model does not mean in vitro gametogenesis. It means in vitro gonadotropin sensitivity.

IVD is the 4-month (in the human) stage of development from primordial follicle to secondary follicle, and IVG is the 11-day stage of sensitivity to gonadotropins that makes the GVs meiotically competent. No prior hormonal stimulation is administered. Any oocytes that advance in culture to mature MII stage from GV (usually about 35%) are then vitrified in standard fashion [2, 3, 5, 6, 24, 25]. The reason why all centers doing IVM get this average of 35% maturation is explainable by understanding IVD and IVG. We noted no difference in results whether the culture media was Quinn’s Cleavage, Sage IVM, or Medicult IVM media. There was also no difference in results whether HCG concentration was either extreme of 10 mIU/ml or 1000 mIU/ml (Tables 1, 2). Poor results were only obtained if the cumulus was stripped immediately, or if the patient had undergone previous chemotherapy, or if she was pre-pubertal. An intact cumulus is essential, but a variety of ordinary medium was used because of our previous results with in-vitro gametogenesis demonstrating the stages of IVD (in vitro development), IVG, (in vitro gonadotropin sensitivity), and IVM (LH or HCG induced). We postulated that many (35%) of these germinal vesicles would already have completed the full phases of IVD and IVG in vivo in the ovary. We thus suspected and demonstrated that no special IVM media would be needed and that almost any concentration of HCG would suffice [4] (Tables 1, 2).

**Table 1:** Patients with no prior chemotherapy who were post-pubertal

Patient age	Reason for cryopreservation	Culture media base used	FSH/HCG	Number of CCs	M II oocytes frozen	IVM rate	Total OTF pieces
27	Large B cell lymphoma of the cervix	Quinn’s Cleavage	75mlU/ml FSH+10mlU/ml HCG	25	12	48%	21
33	Triple negative breast cancer: BRCA positive	Quinn’s Cleavage	75mlU/ml FSH+1000mlU/ml HCG	11	(cumulus 1 stripped immediately)	9%	17
39	Fertility preservation (social)	Medicult IVM	75mlU/ml FSH+1000mlU/ml HCG	3	2	67%	17
17	Large B cell lymphoma	Medicult IVM	75mlU/ml FSH+1000mlU/ml HCG	10	2	20%	20
14	Ewing sarcoma	Quinn’s Cleavage	150mlU/ml FSH+20mlU/ml HCG	34	10	29%	21
30	Fertility preservation for Turner’s syndrome daughter	Quinn’s Cleavage	75mlU/ml FSH+10mlU/ml HCG	24	9	38%	10
18	Breast cancer	Quinn’s Cleavage	75mlU/ml FSH+10mlU/ml HCG	8	2	25%	16
33	Breast cancer	Quinn’s Cleavage	75mlU/ml FSH+1000mlU/ml HCG	26	5	19%	10
25	Breast cancer	Sage IVM	75mlU/ml FSH+1000mlU/ml HCG	14	5	36%	10
14	Non-Hodgkin’s lymphoma	Sage IVM	75mlU/ml FSH+100mlU/ml HCG	37	14	38%	20
19	Hodgkins lymphoma	Quinn’s Cleavage	75mlU/ml FSH+100mlU/ml HCG	40	14	35%	22
13	Rhabdomyosarcoma	Quinn’s Cleavage	75mlU/ml FSH+100mlU/ml HCG	25	14	56%	16
22	Fertility preservation for premature ovarian failure sister donor	Quinn’s Cleavage	75mlU/ml FSH+100mlU/ml HCG	10	4	40%	22 (d)
20	Fertility preservation premature ovarian failure sister recipient	Quinn’s Cleavage	75mlU/ml FSH+100mlU/ml HCG	34	14	41%	22 (r)
23	Breast cancer	Quinn’s Cleavage	75mlU/ml FSH+100mlU/ml HCG	40	10	25%	33
33	Breast cancer	Quinn’s Cleavage	75mlU/ml FSH+100mlU/ml HCG	3	1	33%	9
41	Breast cancer	Quinn’s Cleavage	75mlU/ml FSH+100mlU/ml HCG	5	2	40%	13
33	Rhabdomyosarcoma	Quinn’s Cleavage	75mlU/ml FSH+100mlU/ml HCG	18	6	33%	24
36	Premature ovarian failure	Quinn’s Cleavage	75mlU/ml FSH+100mlU/ml HCG	20	7	35%	12
20	Hodgkins lymphoma	Quinn’s Cleavage	75mlU/ml FSH+100mlU/ml HCG	15	3	20%	22

Average IVM Rate 34% M II’s vitrified

Average number ovary pieces vitrified 18

**Table 2:** No CCs were found in patients with prior chemotherapy or who were pre-pubertal

IVM patients with prior chemotherapy or pre-pubertal								
Patient age	Reason for cryopreservation	Prior chemotherapy	Culture media base used	FSH/HCG	Number of CCs	M II oocytes frozen	IVM Rate	Total OTF pieces
20	ALL	Yes	Unknown	Unknown	0	0	#	11
41	Breast cancer	Yes	Unknown	Unknown	0	0	#	22
9	Gonad blastoma and dysgerminoma	Yes	Sage IVM	75mlU/ml FSH+10mlU/ml HCG	0	0	#	6
38	Breast cancer	Yes	Quinn’s cleavage	75mlU/ml FSH+100mlU/ml HCG	0	0	#	24
2	Turner’s syndrome	No	Quinn’s cleavage	75mlU/ml FSH+100mlU/ml HCG	1 unknown (suspicious egg)	0	#	2
20	AML	Yes	Quinn’s cleavage	75mlU/ml FSH+100mlU/ml HCG	2	0	#	12
3	Neuroblastoma	Yes	Quinn’s cleavage	75mlU/ml FSH+100mlU/ml HCG	0	0	#	4
4	Stage IV Wilms tumor	Yes	Quinn’s cleavage	75mlU/ml FSH+1000mlU/ml HCG	0	0	#	4
22	Alveolar rhabdomyosarcoma	No	Quinn’s cleavage	75mlU/ml FSH+100mlU/ml HCG	6	0	#	19
17	Desmoplastic small round cell tumor	Yes	Quinn’s cleavage	75mlU/ml FSH+100mlU/ml HCG	0	0	#	8
36	Breast cancer	No	Quinn’s cleavage	75mlU/ml FSH+100mlU/ml HCG	3	0	#	16
33	Breast cancer	Yes	Quinn’s cleavage	75mlU/ml FSH+100mlU/ml HCG	0	0	#	24
4	Stage IV Wilms tumor	Yes	Quinn’s cleavage	75mlU/ml FSH+100mlU/ml HCG	0	0	#	6
9	B cell ALL	Yes	Quinn’s cleavage	75mlU/ml FSH+100mlU/ml HCG	0	0	#	4
36	Breast cancer	Yes	Quinn’s cleavage	75mlU/ml FSH+100mlU/ml HCG	0	0	#	12
11	Wilms tumor	Yes	Quinn’s cleavage	75mlU/ml FSH+100mlU/ml HCG	0	0	#	13
12	Neuroblastoma	Yes	Quinn’s cleavage	75mlU/ml FSH+100mlU/ml HCG	0	0	#	6
19	ALL	Yes	Quinn’s cleavage	75mlU/ml FSH+100mlU/ml HCG	0	0	#	21
21	Turner’s syndrome	No	Quinn's cleavage	75mlU/ml FSH+100mlU/ml HCG	0	0	#	12

Average number of M IIs: 0

Average percentage of CCs to M IIs: 0

**Fig. 4:**
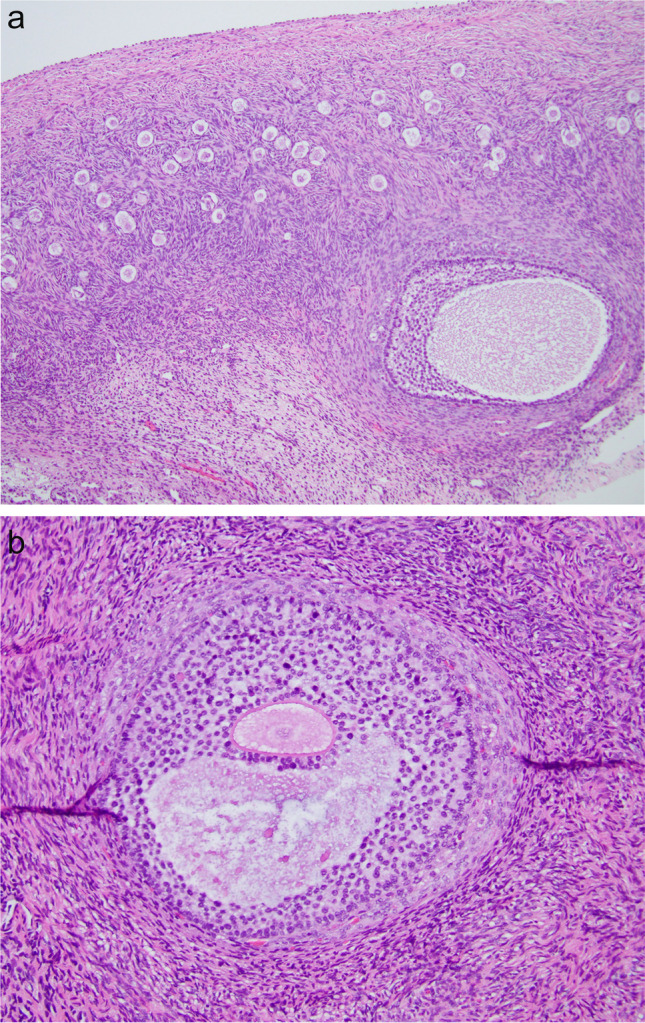
**a** Histologic view of initially developing recruited follicle exiting ovarian cortex. **b** A histologic view of germinal vesicle follicle about to exit cortex

Note that with needle aspiration, one is not able to obtain the small follicles that are right at the cortico-medullary junction (Fig. 4a, b). Yet these are likely to be the best follicles for IVM because of the location of FSH in the granulosa cells [26–28]. The FSH concentration is important for sensitizing LH receptors for IVM. The FSH concentration is highest in the mural granulosa cells of the follicle, and not in the corona in the granulosa cells by a ratio of 100:1. A high concentration of FSH in mural cells is only found in the smaller CCs (cumulus complexes) closer to the outer rim of the cortex and these smaller CCs nonetheless have large 100 microns GVs that are ready for IVM. So not only is surgical dissection more likely to successfully retrieve GVs but it is also more likely to retrieve the LH-sensitive mural granulosa cells.

## Maturation of Cumulus Complexes Recovered in the Media After Cortico-medullary Dissection

The number of CCs recovered can vary widely, between 3 and 80 or more cumulus complexes, depending on the age of the women and whether there had been any prior chemotherapy. For IVM as we have reported, a total of 34 females underwent oophorectomy for ovary tissue freezing and IVM [4]. In 14 of those patients there was prior chemotherapy or they were pre-pubertal and there were no CCs found. In the remaining 20, IVM was performed. [4] (Tables 1, 2). The rate of maturation to an MII oocyte (Fig. 3a–d) varied from a low of 19% to a high of 56% (average 35%) Once exception is where the cumulus was dissected. The average values from other centers publishing on IVM has also varied between 30 and 39% [2, 3, 8, 9, 11, 12, 29–31]. Maturation of germinal vesicle to MII oocytes is detected between 24 and 48 h of exposure to the HCG-containing media. Interestingly, Previous research indicated that the number of mature oocytes we obtain from IVM is similar to what would be obtained from ovarian stimulation [2]. A variety of media and gonadotropin concentrations were intentionally used in light of the mechanisms of in vitro oogenesis, in order to see if this new understanding of ovarian function could allow for a simplification of IVM [4, 8–12, 14].

As these results (and those reported by other groups) with IVM demonstrate, one might consider with cancer patients the option to dispense with ovarian stimulation, and go right to oophorectomy, with no delay in cancer treatment. There might be no need for stimulation to obtain many mature oocytes [2, 3, 5, 6, 25]. Mature oocytes may simply be directly obtained (as an extra benefit) from the excised ovarian tissue before cryopreservation of the tissue. Other groups have preceded our current efforts at IVM from excised ovarian tissue and achieved pregnancy with healthy infants [1, 3, 5–7, 25].

## Mechanism In vivo and In vitro of Oocyte Maturation

What is most fascinating about this very simple IVM is why does it work? It might at first seem puzzling why IVM from ovarian tissue suddenly seems so easy at many different centers, when it has been so difficult in the past. There are several potential reasons for this. First, we were not trying to mature primordial follicles, although that should become possible in the future with the “8 core genes” approach, which works well in mice [14]. However, culturing germinal vesicle oocytes “GVs” that have already become meiotically competent by in vivo “IVD” and in vivo “IVG” should not be expected to be difficult. For example, in a young woman on average every single day about 30 GVs will leave the inert IVD phase, and will become sensitive to gonadotropin. About 120 GVs will have been exposed to gonadotropin for more than 7, and less than 12 days IN VIVO. Of course, one must obtain many CCs containing GVs for this to be practical, and it is far easier to obtain many germinal vesicle oocytes with cortical dissection rather than with a needle. Figure 4a and b demonstrate clearly why dissection of the cortico-medullary junction is so much more effective than trying to obtain these CCs from tiny follicles than with a needle. The results of in vitro gametogenesis and IVM also reveal the limited role of the ovulatory cycle and ovarian stimulation in oocyte development other than to remove the oocyte from the ovary [9, 10, 29], 2013; [2, 11, 30, 31].

**Fig. 5:**
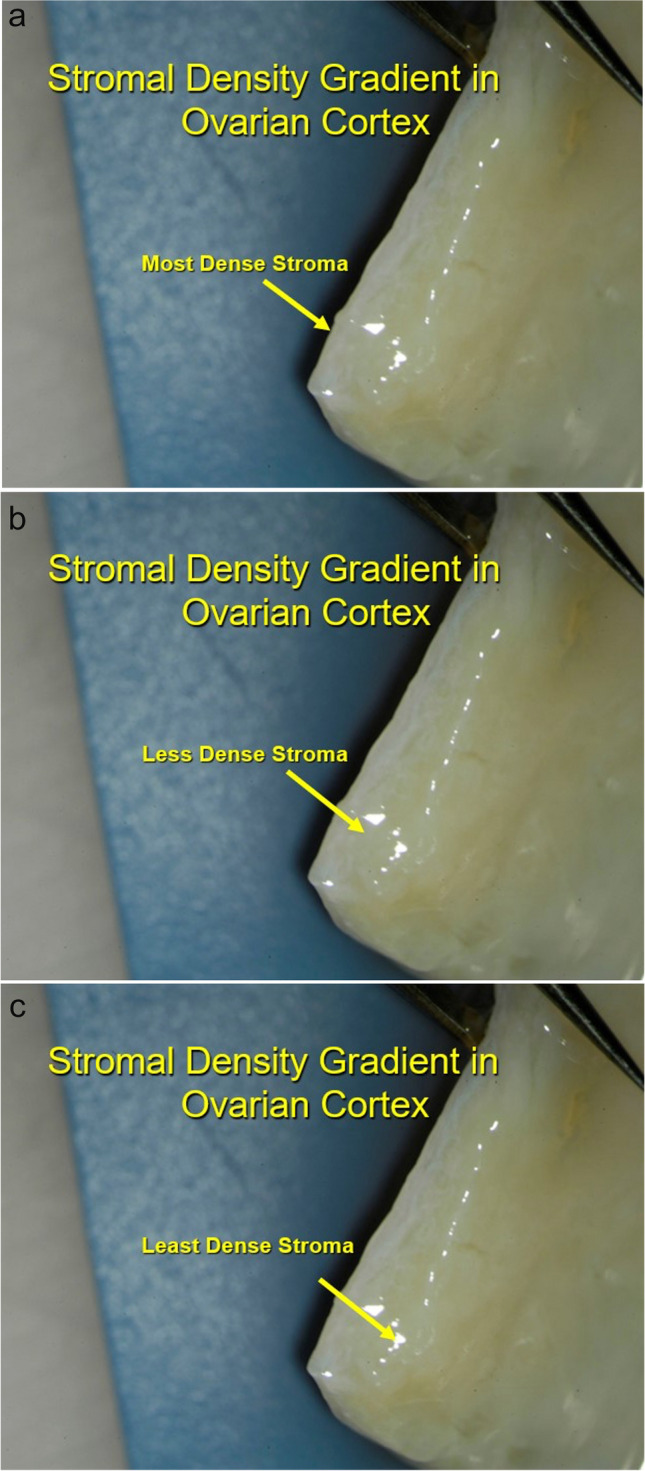
**a** Surgical view of densest outer cortex. **b** Surgical view of less dense internal cortex. **c** Histologic view of innermost least dense ovarian cortex

**Fig. 6:**
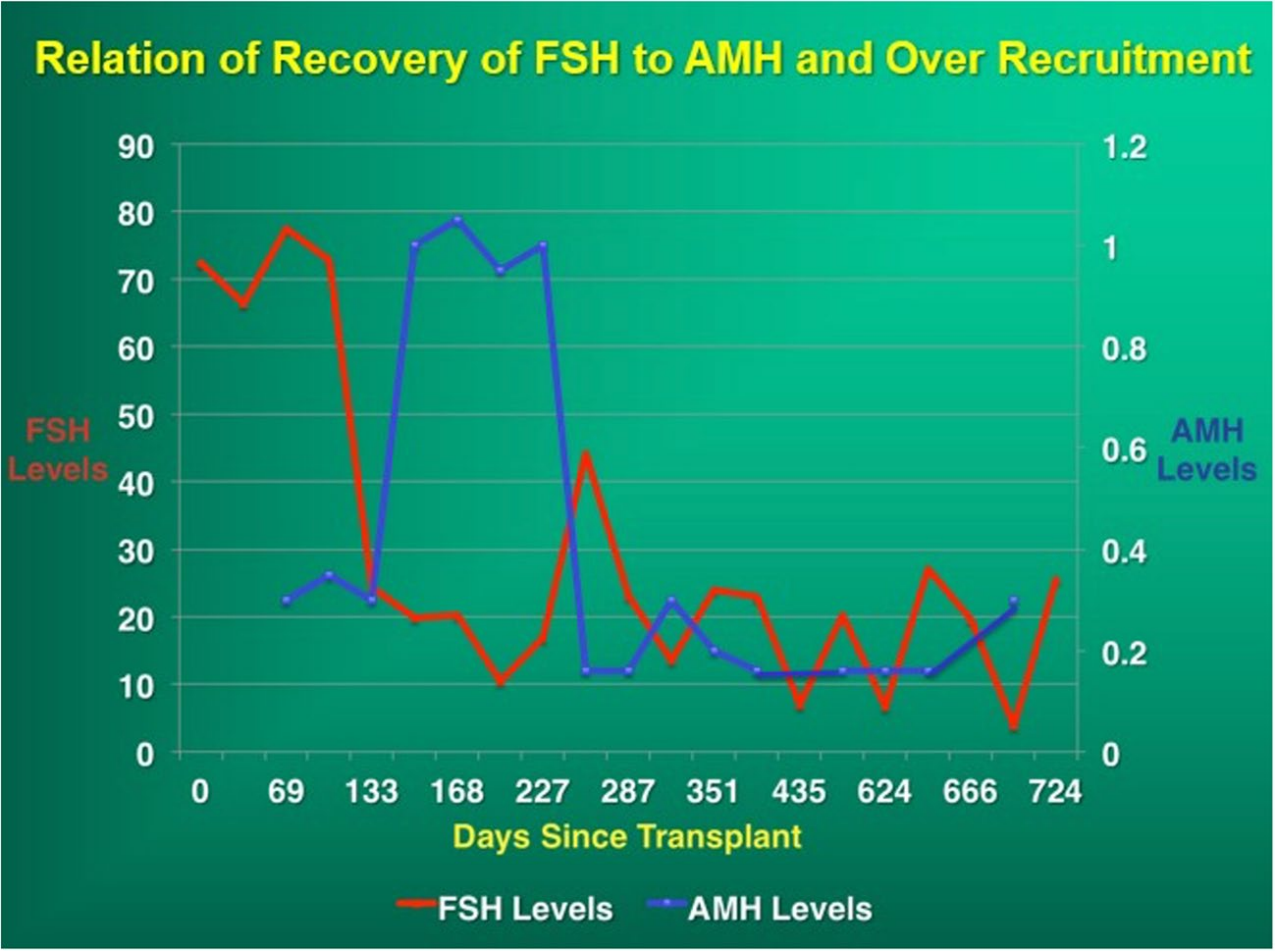
Relation of recovery of FSH to AMH and Over Recruitment. There is a 4-–5-month period after ovary tissue transplant where FSH remains high and AMH is undetectable. Then when the FSH starts coming down, the AMH rises indicating that primordia follicles have finally differentiated into gonadotropin sensitivity, releasing estrogen, inhibiting FSH, and secreting AMH. However, the AMH then goes up very high for 4–6 months indicating over-recruitment from primordial follicle 4.5 months earlier. Because of subsequent depletants, the AMH then goes down. It stays down for many years because of the well-known phenomenon that as the ovarian reserve goes down, the rate of primordial follicle recruitment also goes down. So despite low AMH, these grafts (only ½ of an ovary) last for 8–10 years

Intrinsic tissue pressure (along with eight “core genes”) has been shown with in-vitro gametogenesis to be the initiating mechanism at work to control primordial follicle recruitment and development to antral follicle status [2, 3, 5, 6, 11, 12, 23, 25, 32, 33]. The in vivo cortical tissue pressure gradient is a major regulator of primordial follicle recruitment and ovarian longevity [22, 34, 35] (Fig. 5a–c). This pressure theory is supported clinically by the changes in AMH and FSH observed and previously reported and discussed in fresh and frozen ovary tissue transplants [22, 32, 36] (Fig. 6). As the FSH comes down to normal levels 4–5 months after the transplant, at the same moment AMH begins to rise from zero up to very high levels, and then 9 months later comes down again to low levels, indicating an over-recruitment of primordial follicles because of tissue pressure reduction at the time of transplant. Then despite low AMH, the graft continues to function for many years because as ovarian reserve decreases, follicle recruitment also decreases.

**Fig. 7:**
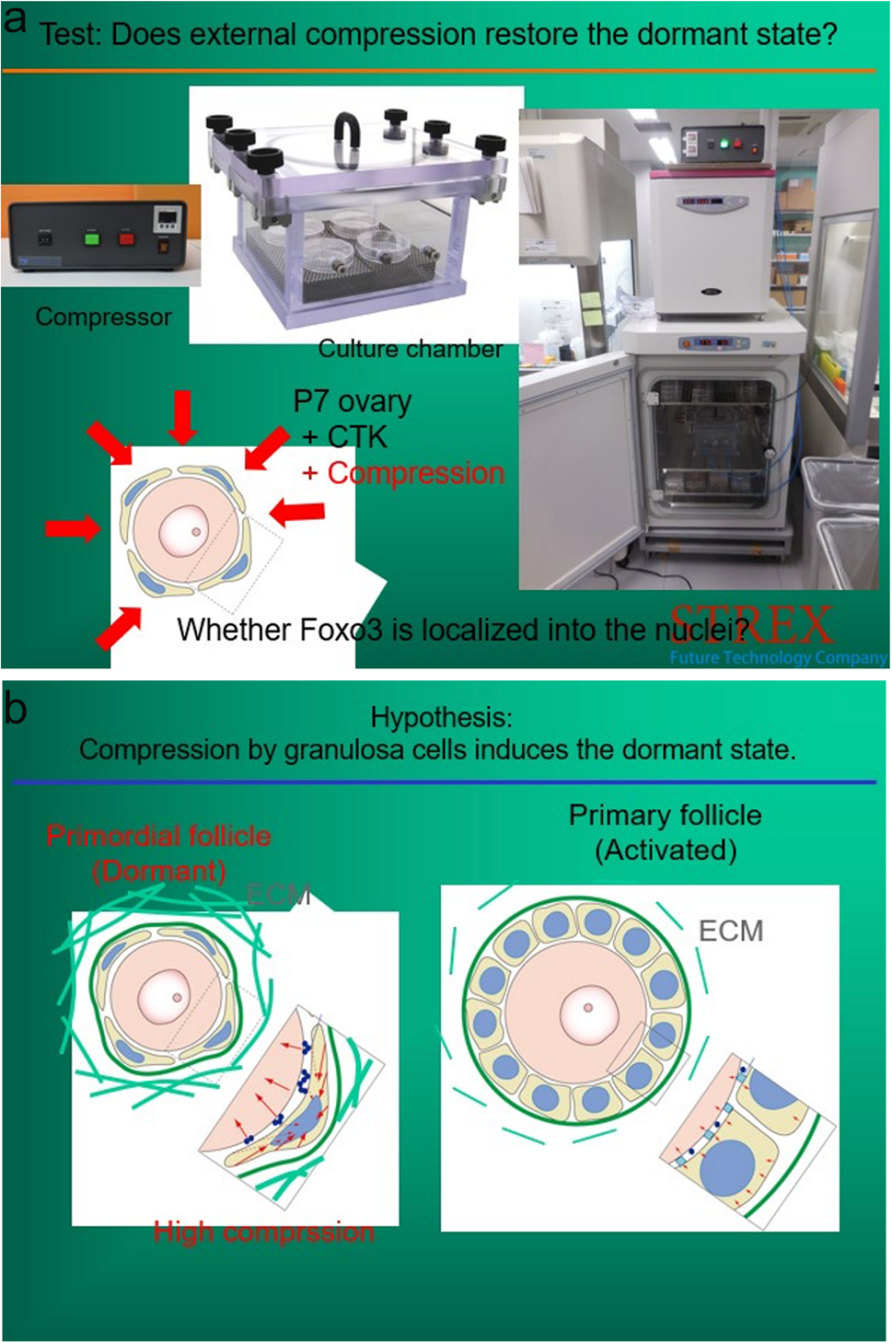
**a** Incubation of in vitro generated oocytes at high atmospheric pressure arrests meiotic progression [12]. **b** Diagram of compression of cumulus cells causing dormancy in oocytes [12]

Primordial follicle arrest in the highly compact ovarian cortex is thought to be a possible key to saving the oocyte from disappearing after the fetal initiation of meiosis and prevents its continuation all the way through meiosis and subsequent apoptosis [8–12]. It may also be a key to the gradual recruitment every month of a limited number of oocytes in the adult to develop over 4 months into gonadotrophin-sensitive antral and graafian follicles, which spares the resting oocytes from sudden total depletion [23] (Fig. 5a–c). The IVD stage in mice of 3 weeks from primordial follicle to gonadotropin sensitive secondary follicle is all controlled by decreasing pressure gradient and just eight “core genes.” In humans this stage is much longer (4 months). In culture systems for in vitro gametogenesis (as opposed to in vivo), oocytes developed from IPS cells will differentiate quickly through IVD. However, if these cultures are performed in a high pressure incubator, the nuclei start rotating and development is arrested. Then when placed into a normal low pressure environment, the nuclei stop rotating, and the oocytes resume developing [12] (Fig. 7a–c).

Perhaps the most striking scientific postulate about ovarian function resulting from this series is the high rate of maturation of germinal vesicle oocytes from ovary tissue to MII in less than 2 days, with no ovarian stimulation. The success of IVM did not correlate with the specifics of the media used or the concentration of gonadotrophin. The success of IVM appears to be intrinsic to the cumulus and they have already achieved meiotic competence by in vivo exposure to the “core genes” (IVD), and to in vivo FSH (IVG) [14].

On average the ovarian cortex of a young woman contains about 200,000 oocytes. Every month, about 1000 are recruited from “resting” follicles in the cortex, and they require 4–5 months (IVD) thereafter to become sensitive to gonadotrophins and enter the ovulatory cycle. In-vitro gametogenesis studies in mice have termed this phase “IVD,” i.e., in vitro differentiation to germinal vesicle stage. This phase, also called “PPT” (primordial to primary follicle transition) represents the non-gonadotropin-sensitive growth from either recruited oocytes or pluripotent stem cell to germinal vesicle oocytes (which are now sensitive to gonadotrophin). IVD requires 3 weeks in mice, but closer to 4 months in humans [8, 10, 22, 32, 34, 37]. IVD in vivo is a constantly occurring process, as also is IVG. There is a continuous exposure to FSH of IVG-ready oocytes in the intact in vivo ovary. The IVG process usually takes about 8–11 days in both mice, and presumably in humans. Oocytes that have completed IVG but then are not immediately exposed to HCG in vitro, or to the ovulatory LH surge in vivo, become post-mature and gradually degenerate. It is the population of oocytes retrieved from the dissected ovarian cortex that have just recently completed IVG (35%) that mature readily with exposure to HCG in a variety of concentrations in ordinary culture media.

**Fig. 8:**
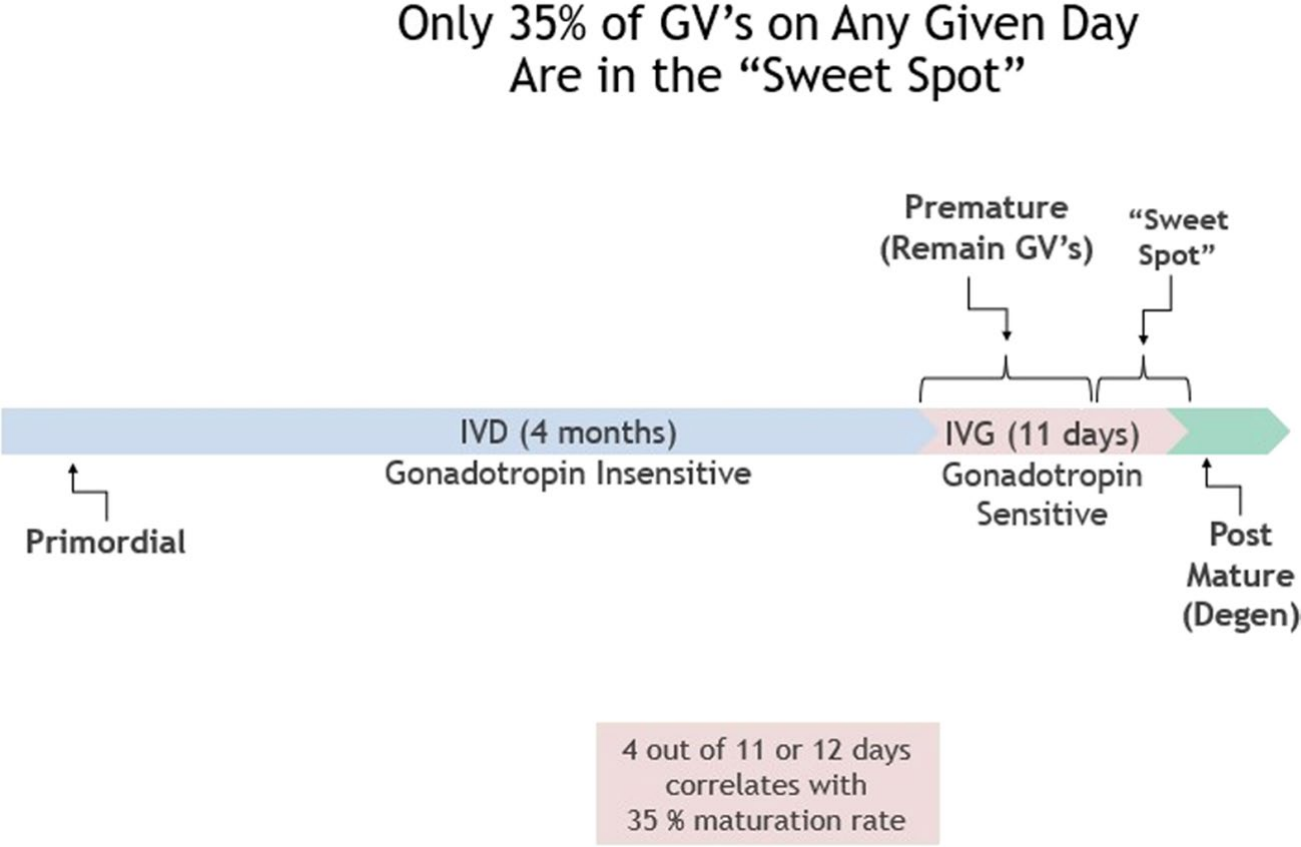
Diagram explaining in vitro maturation (IVM) of oocytes from ovarian tissue in human, based on mouse model of in vitro oogenesis

It should be noted from Fig. 8, the most important figure in this paper, that on any given day there are about 35% of oocytes that have undergone at least 7–8 days of IVG, but also less than 12 days where degeneration occurs. There is thus a “sweet spot” between 7 days and 11 days of gonadotropin exposure where GVs can mature in vitro and not degenerate (Fig. 8). Less than seven days means not enough exposure to in vivo gonadotropin to become sensitive to HCG or LH. More than 11 days exposure of in vivo means they are post-mature and they will degenerate. That is why on any given day 35% of GVs will mature to M2 if exposed to HCG. If exposure to FSH after completing IVD is less than 7-8 days the GV will not mature in HCG. If exposure to FSH has been greater than 12 days, the GV will just degenerate.

## Recruitment from Primordial Follicle to GV Oocyte

The phase referred to as “IVG,” i.e., gonadotrophin (FSH)-induced meiotic competence of germinal vesicle oocytes, usually requires 7–11 days, remarkably similar to ovarian stimulation in human IVF. The unstimulated ovary has already been exposed to FSH in vivo. Many (average 35%)of the germinal vesicle oocytes (in cumulus complexes) recovered from the ovarian dissection have already gone through the “IVD” and “IVG” phases in vivo, and therefore are meiotically competent, having already had adequate exposure to endogenous FSH. Therefore just 1–2 days of exposure to LH or HCG is all that is needed for these specific germinal vesicle oocytes to develop to mature MII oocytes. What has previously not been understood is how the primordial follicles are recruited and nourished to go through IVD and become gonadotropin sensitive GVs.

**Fig. 9:**
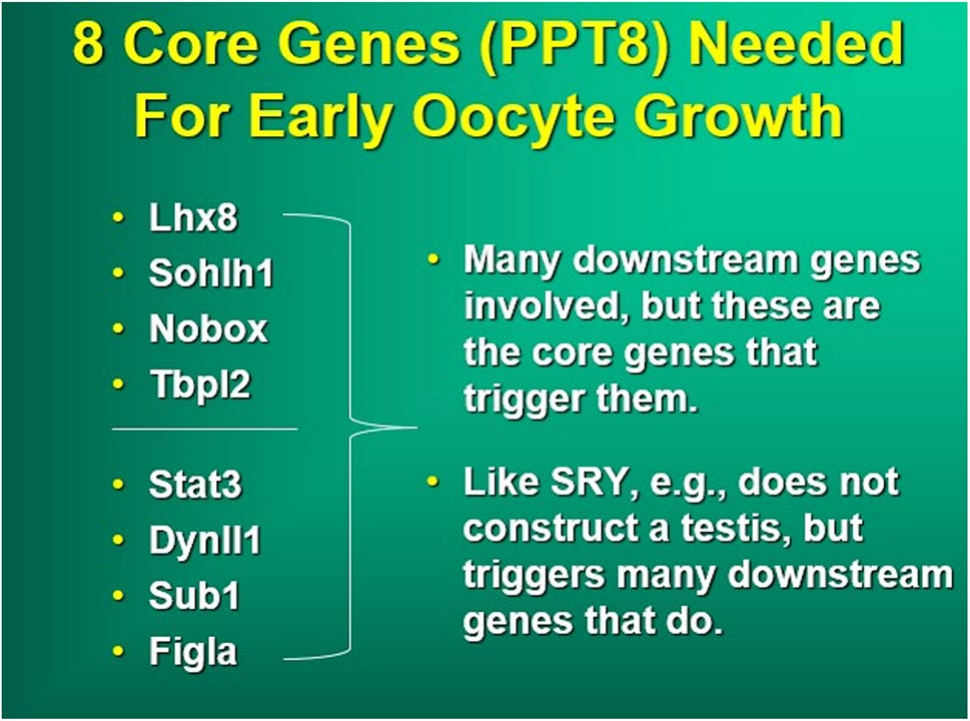
Only eight genes in the mouse are necessary for primordial follicle recruitment to competent secondary follicles

The eight “core genes” in mice are all that are needed to convert stem cells to oocytes (Fig. 9). However, the oocytes that develop directly from stem cells are not fully competent. Full competence requires culture with fetal granulosa cells, which can also be produced from stem cells using a more complex culture system [13]. However, if these eight core genes are used to recruit from the primordial follicles (that have already been exposed to granulosa cells when the ovary was just fetal), then the resulting M2 oocytes are fully functional and can result in normal offspring.

Without the dense pressure of the ovarian cortex in the fetus, which induces the formation of primordial follicles, fetal oocytes would continue in meiosis and be completely depleted by birth [8–10, 38–40]; Hikabe [22, 32, 41–43]. Nagamatsu and colleagues demonstrated that dense cortical tissue pressure caused oocyte nuclei to rotate, and holds the primordial follicles in arrest [12]. As they encounter less tissue pressure internally, the rotation stops, and the primordial follicles are recruited [12] (Fig. 7a, b). These eight “core genes” can also recruit stem cells or IPS (induced pluripotent stem cell) cells to transform all the way to MII oocyte-like cells that are not competent but recruitment from primordial follicles (from ovarian tissue) results in normal, competent oocytes. [14, 44–46]; Winkler-Crepaz [32, 47, 48]. Therefore, with IVM it should become possible to obtain normal MII oocytes from ovarian tissue. However, for competent oocytes to develop directly from IPS cells in vitro, will require co-cultures with fetal (not adult) granulosa cells. That will require transforming IPS cells in a separate culture system using the Yoshino approach into species-specific fetal granulosa cells [13].

## Conclusions

The only way to be certain of the functional competence any of these MII oocytes is if live births can be obtained. Several reports have already shown this to be the case with IVM from ovary tissue [1–3, 5, 6, 25]. What is a new and perhaps shocking suggestion is the relative ease with which this can be achieved using ordinary media. This would be expected from our group’s previously published studies of in-vitro gametogenesis from stem cells in mice. It is easy to collect many germinal vesicle oocytes from the tiny follicles at the cortico-medullary junction when you have the ovary in hand instead of using a needle, and their intrinsic 30–40% meiotic competence is universal. So why do we even need ovarian stimulation, or even the normal ovulatory cycle? The normal ovulation cycle is not needed for meiotic competence. Ovarian stimulation is only required to allow oocytes to exit the ovary. To apply this simple and robust IVM to women undergoing IVF with ultrasound and needle will require a special technique to aspirate the smallest follicles of 2mm to 6mm [49–51]. It is easy to obtain CCs from these follicles using dissection. It will be more difficult (but possible) using ultrasound guided needle aspiration.

Simple cortical tissue pressure (associated with primordial follicle nuclear rotation) has been found to be a key regulator of primordial follicle arrest, recruitment and ovarian longevity in humans, similar to mice [12]. However, eight “core genes” (in mice) and exit of intranuclear FOX3 are also necessary to allow the primordial follicles to escape arrest and develop to meiotically competent germinal vesicle oocytes. The ability of ovarian tissue germinal vesicle oocytes to undergo normal IVM indicates that the normal ovulatory cycle and ovarian stimulation is not necessary for oocyte maturation. Eventually, we can project that all that will be needed to make competent MII oocytes in our patients of any age is a skin biopsy as a source for IPS cells. We already can make competent human PGCs from IPS cells. Now we have to make fetal granulosa cells from IPS cells.

## Data Availability

The datasets used and/or analyzed during the current study are available from the corresponding author on reasonable request.
